# Availability and cost of major and first-line antiepileptic drugs: a comprehensive evaluation in the capital of Madagascar

**DOI:** 10.1186/s40064-016-3409-5

**Published:** 2016-10-06

**Authors:** Jeremy Jost, Adeline Raharivelo, Voa Ratsimbazafy, Mandy Nizard, Emilie Auditeau, Charles R. Newton, Pierre-Marie Preux

**Affiliations:** 1 INSERM, Univ. Limoges, CHU Limoges, Department of Pharmacy, UMR_S 1094, Tropical Neuroepidemiology, Institute of Neuroepidemiology and Tropical Neurology, CNRS FR 3503 GEIST, 2 rue du Docteur Marcland, 87025 Limoges Cedex, France; 2 Hôpital Joseph Raseta Befelatanana, Antananarivo, Madagascar; 3 Department of Psychiatry, University of Oxford, Oxford, UK; 4 KEMRI-Wellcome Trust Programme Centre for Geographical Medicine (Coast), Kenya Medical Research Institute, Kilifi, Kenya

## Abstract

**Background:**

The prevalence of epilepsy is high in Madagascar (23.5/1000), as is the treatment gap (estimated at 92 %). The health system of the country is underfunded; some AEDs are used, and the national drug policy does not encourage price regulation or the administration of generic agents. We conducted a cross-sectional study to assess the availability and cost of solid oral AED formulations in Antananarivo, capital of Madagascar. Data were gathered from all officially registered pharmacies (according to the drug agency list, updated in 2015) by means of telephone interviews lasting no more than 10 min and conducted by a native Malagasy speaker. With regard to other sources (hospitals, illicit sales) data were obtained at specific visits. The study received ethical approval from the Madagascar Ministry of Health.

**Findings:**

A total of 91 of 100 pharmacies (the nine not included were because of an inoperative phone number), two of three public hospitals, and two illegal outlets were investigated. Sodium valproate was available in 84.6 % of the pharmacies, while carbamazepine and phenobarbital were available in 68.1 % and 36.3 % of the pharmacies, respectively, but phenytoin was not available in any supply chain. There were more originator brands than generic formulations, with a higher cost (range 20.3–81.1 %, median 40.7 %) compared to the equivalent generic. The public system had only a very limited choice of AED, but offered the lowest costs. Illicit sources were more expensive by 54.3 % for carbamazepine and 62.5 % for phenobarbital. Concerning the annual cost of treatment, the average percentage of the gross national income per capita based on the purchasing power parity was 29.8 %/19.0 % (brand/generic) for sodium valproate, 16.4 %/7.3 % (brand/generic) for carbamazepine, 8.9 %/5.1 % (brand/generic) for phenobarbital.

**Conclusions:**

The main sources of AEDs were private pharmacies, but the stocks held were low. The financial burden was still important in the capital of Madagascar, mainly the consequence of a highly developed private sector at the expense of the public sector. Although sodium valproate remains the most expensive solution, it still remains the most available instead of phenobarbital. The most striking feature of this study concerns the cost of AEDs in the informal sector, mostly used because they are deemed to provide less costly drugs, the opposite was observed there. The assessment of the cost and availability of medicines was easily and quickly implemented. It provided a relevant focus of the situation in areas difficult to investigate, in terms of road network and geographical situation.

## Background

Epilepsy is a chronic neurological disease affecting more than 70 million people worldwide (Ngugi et al. 2010). Among them, nearly 90 % live in resource-limited settings where problems of accessibility, affordability and availability of antiepileptic drugs are dramatic. In those areas, the treatment gap (proportion of people with epilepsy who require treatment but do not receive it) has been established to be over 75 % (Meyer et al. 2010). Primary healthcare is extremely limited in developing countries, where only a small proportion of major and first-line antiepileptic drugs (AEDs) are commonly used: phenobarbital, carbamazepine, sodium valproate, phenytoin. The burden is even more dramatic in rural areas where in addition to a low availability the sustainability of drugs supply is often not guaranteed (Perucca 2007). This situation could also be seen in developed European countries where the median availability of all type of AEDs (older and newer) is 82 % ranging from 48 to 100 % (Baftiu and Johannessen 2015). Madagascar is not spared, with a high prevalence of epilepsy (23.5/1000) (Ba-Diop et al. 2014). The treatment gap has been estimated to 92 % by an indirect method (based on the drug consumption over the year) and at 32 % by a direct method (based on how many of the detected cases are not receiving treatment in a prevalence study) (Meyer et al. 2010; Ratsimbazafy et al. 2011; Kale 2002). The health system of the country is underfunded; some antiepileptic drugs (AEDs) are used, but the national drug policy does not encourage price regulation or the administration of generic agents. The total health expenditure in Madagascar was 3.04 % of the Grow Domestic Product which was in 2015 and per capita around 1439 current international $, adjusted on the purchasing power parity (World Bank data, 2014). Against this background, we conducted a cross-sectional study to assess the availability and cost of solid oral AED formulations in Antananarivo, capital of Madagascar, which had a population of 2.2 million in 2014.

## Methods

This study was performed as an ancillary study of a project aiming at assessing the quality of solid oral AEDs formulations in sub-Saharan Africa, in twelve countries and in all kind of supply chain (official and illegal one). Information on availability and cost was gathered from all officially registered pharmacies (according to the drug agency list, updated in July 2015) by means of telephone interviews lasting no more than 10 min and conducted by a native Malagasy speaker. With regard to other type of supply chain (hospitals, illicit sales) data were obtained at specific visits. The study received ethical approval from the Madagascar Ministry of Health.

## Results

A total of 91 of 100 pharmacies (the nine not included were because of a wrong/inoperative phone number), two of three public university hospitals, and two illegal outlets were investigated. Sodium valproate was available in 84.6 % of the pharmacies, while carbamazepine and phenobarbital were available in 68.1 and 36.3 % of the pharmacies, respectively, but phenytoin was not available in any supply chain. Availability and costs by AED and dosage are detailed in Table 1. There were more originator brands than generic formulations (e.g. sodium valproate 200 mg, 84.2 % for originator brand compare to 4.4 % of generic), with a higher cost (range 20.3–81.1 %; median 40.7 %) compared to the equivalent generic. The public system had only a very limited choice of AED, but offered the lowest costs in the official system (10.0 % lower for sodium valproate, 36.4 % for carbamazepine and 43.7 % for phenobarbital). Illicit sources were more expensive by 54.3 % for carbamazepine and 62.5 % for phenobarbital; this trend was less marked for sodium valproate. All sources had only one or two boxes of each molecule available. Concerning the annual cost of treatment, the average percentage of the gross national income per capita based on the purchasing power parity (current international $) was 29.8/19 % (brand/generic) for sodium valproate, 16.4/7.3 % (brand/generic) for carbamazepine, 8.9/5.1 % (brand/generic) for phenobarbital.

**Table 1:** Availability and costs by AED and dosage, in Antananarivo

Dosage	% of availability in pharmacy (n = 91)	% of availability in public hospital (n = 2)	% of availability in illicit circuit (n = 2)	Average price (±sd) per unit in pharmacy $US	% of difference of the average price compared to pharmacy (H: hospital; I: illicit)	Annual cost of treatment^b^ (1 year = 365.25 day), $US	% of the GNI per capita, PPP (current international $)^c^	Ratio brand/generic
*AI*								
Sodium valproate								
Generic								
200	4.4	0.0	0.0	0.094 (±0.014)		274.7	19.6	
500	2.2	0.0	0.0	0.235^a^		257.5	18.4	
500 ER	0.0	0.0	0.0	NA		NA	NA	
Originator brand								
200	84.6	0.0	50.0	0.131 (±0.012)	I: +12.7 %	382.8	27.3	×1.39
500	80.2	0.0	50.0	0.295 (±0.012)	I: −11.9 %	323.2	23.1	×1.26
500 ER	63.7	100.0	50.0	0.497 (±0.011)	H: −9.5 %; I: −25.6 %	544.6	38.9	
Carbamazepine								
Generic								
200	63.7	50.0	100.0	0.032 (±0.013)	H: −37.5 %; I: +54.3 %	46.8	3.3	
400	0.0	0.0	0.0	NA		NA	NA	
200 ER	2.2	0.0	0.0	0.130 (±0.014)		189.9	13.6	
400 ER	0.0	0.0	0.0	NA		NA	NA	
Originator brand								
200	68.1	0.0	0.0	0.169 (±0.020)		246.9	17.6	×5.28
400	1.1	0.0	0.0	0.167^a^		122.0	8.7	
200 ER	58.2	0.0	0.0	0.185 (±0.018)		270.3	19.3	×1.42
400 ER	28.6	0.0	0.0	0.380 (±0.043)		277.6	19.8	
Phenobarbital								
Generic								
10	1.1	0.0	0.0	0.033^a^		120.5	8.6	
50	1.1	50.0	100.0	0.030^a^	H: −33.3 %; I: +62.5 %	21.9	1.6	
100	0.0	0.0	0.0	NA		NA	NA	
Originator brand								
10	1.1	0.0	0.0	0.076^a^		277.6	19.8	×2.30
50	30.8	0.0	0.0	0.062 (±0.006)		45.3	3.2	×2.07
100	36.3	0.0	0.0	0.144 (±0.008)		52.6	3.8	

1 $US = 3307.58 Ariary

*AI* active ingredient, *NA* not available, *ER* extended-release, *GNI* gross national income, *PPP* purchasing power parity, *I* illicit, *P* pharmacy, *H* hospital

^a^Only one sample has been listed, the mean and standard deviation were not calculable

^b^Usual daily dose for a 70 kg patient weight (mg): VPA: 1500 mg/day; CBZ: 800 mg/day; PB: 100 mg/day

^c^GNI per capita PPP in Madagascar (2014) = 1400 $US (World Bank Data)

## Discussion

The main sources of AEDs were private pharmacies. Three of the four major AEDs were available, but the stocks held were low. The financial burden for people with epilepsy was still important in the capital of Madagascar. This was mainly the consequence of a very developed private sector at the expense of the public sector, without any price regulation. Originator brand remained more expensive than generic, ranging from 1.3 to 5.3 higher. This result was less dramatic than data observed in a large survey conducted in 46 countries, where originator brand prices were about 30 times higher (Cameron et al. 2012). Furthermore, although sodium valproate remains the most expensive solution, it still remains the most available instead of phenobarbital. However, the most striking feature of this study concerns the cost of AEDs in the informal sector: patients use these outlets mostly because they are deemed to provide less costly drugs, but the opposite was observed in Madagascar. This results must be confirmed by further investigations in other sites but the illicit supply chain is difficult to assess. Selling points are often not officially known, and these information are often obtained by word of mouth. The methodology to measure the cost and availability of medicines by phone call, was easily and quickly implemented. The main limit of this method is due to the cross-sectional assessment, that provide information in a given position at a given time, that could not be the real availability at any moment. Anyway, it can provide a relevant and a comprehensive focus of the situation in a very large and crowded area with many points to be investigated and with a heavily congested and practically unusable road network. This epidemiological assessment provide data that could contribute to address the financial burden for people with epilepsy in the capital of Madagascar.
